# Novel Alginate-, Cellulose- and Starch-Based Membrane Materials for the Separation of Synthetic Dyes and Metal Ions from Aqueous Solutions and Suspensions—A Review

**DOI:** 10.3390/ma18245495

**Published:** 2025-12-06

**Authors:** Małgorzata A. Kaczorowska

**Affiliations:** Faculty of Chemical Technology and Engineering, Bydgoszcz University of Science and Technology, Seminaryjna 3, 85-326 Bydgoszcz, Poland; malgorzata.kaczorowska@pbs.edu.pl

**Keywords:** water resources pollution, synthetic dyes, metal ions, polysaccharide membranes, innovative materials

## Abstract

Pollution of water resources with hazardous substances of anthropogenic origin (e.g., synthetic dyes, heavy metal ions) is currently one of the most important environmental issues, and the development of not only effective and economical but also eco-friendly methods of removing these substances from aqueous solutions is one of the greatest challenges. Among the various separation methods, techniques based on the utilization of different types of polymer membranes have gained increasing interest due to their usually high efficiency, the materials’ stability and reusability, and the possibility of using “green” components for their formation. Recent research efforts have been concentrated, inter alia, on the application of natural polysaccharide polymers (e.g., cellulose, alginates, starch, cyclodextrins) and their derivatives to produce well-performing membranes. Appropriately composed polysaccharide-based membranes under optimal process conditions enable effective separation of dyes, salts, and metal ions (e.g., often with a rejection rates of >95% for dyes and metal ions and <7% for salts). This review concerns the latest developments in the formation and utilization of novel polysaccharide-based membranes for the separation of synthetic dyes and metal ions from aqueous solutions and suspensions, with emphasis on their most important advantages, limitations, and potential impact on the environment and sustainability.

## 1. Introduction

Due to decreasing freshwater resources, pollution of water reservoirs related to human activities and increasing demand for good-quality water, appropriate treatment of wastewater generated by various industries is currently a global challenge [1]. Industrial wastewater is typically a complex mixture containing various compounds that can pose a risk to the health of humans and ecosystems. For example, effluents originating from the textile dyeing industry, which is an important part of the global economy and one of the main sources of environmental pollution, contain not only dyes but also heavy metals and organic solvents. The diverse composition of such wastewater and the nature of the pollutants, many of which are non-degradable and toxic, mean that the application of traditional treatment methods often does not allow for the satisfactory removal of all hazardous substances and is usually associated with the generation of various by-products, which are also not indifferent to the environment (e.g., sewage sludge that requires special treatment) [2,3].

In general, in wastewater treatment, a variety of processes are used and their selection depends on the nature (e.g., chemical composition, presence of certain microorganisms, pH, temperature) and quantity of wastewater generated. In the case of treating wastewater from the dyeing industry, physicochemical, biological, and electrochemical methods as well as advanced oxidation processes are usually utilized. Common methods used include coagulation, adsorption, flocculation, filtration, and combinations of various techniques, enabling an increase in the efficiency of treatment processes [4]. In addition to using well-established solutions, new, more efficient, and environmentally friendly wastewater treatment methods are being systematically sought, the use of which allows for effective separation of contaminants and leads to fewer by-products [3].

Furthermore, currently, a lot of attention is gained by sustainable wastewater treatment strategies focusing not only on removing specific pollutants from wastewater but also taking into account the optimization of resource recovery (complex wastewater and sewage sludge may be a potential resource pool) and energy efficiency [2,5,6,7]. Conventional separation methods usually fail to achieve all of the important goals during wastewater treatment, including effective removal of specific pollutants, minimal hazardous waste generation, environmental safety of treatment techniques, low energy consumption, and the recovery of all valuable components.

Among the alternative methods used to remove various pollutants from aqueous solutions, which have been gaining increasing interest in recent years, are various membrane processes (MPs), usually characterized by adequate effectiveness, simplicity, modularity, and energy efficiency [8]. Moreover, membrane technology has been recognized as having the potential to bridge the economic and sustainability gap, due to, inter alia, relatively low chemical consumption and environmental safety [9]. Membranes, which act as barriers separating two phases by selectively restricting the movement of components through them, can be classified in various ways. Due to their structure, they can be divided into isotropic (characterized by a uniform composition and physical structure) and anisotropic (inhomogeneous across the entire surface, consisting of different layers with varying structures and compositions). Based on the character of materials used to produce them, they can be classified as organic (mainly made of synthetic organic polymers), inorganic (e.g., formulated using ceramics, metals), or composite and hybrid membranes (containing various organic and inorganic components) [9]. Whereby, this is a basic division, as each of the groups can be divided into several subgroups, e.g., in the ceramic membranes group, alumina, zirconia, and titania-based membranes can be distinguished [10]. Another division of membranes can be based on their overall geometry (e.g., flat sheet, spiral wound, tubular, hollow fiber, nanofibrous membranes) [11].

The classic division of membrane separation technologies distinguishes four main types of membranes: reverse osmosis (RO), nanofiltration (NF), ultrafiltration (UF), and microfiltration (MF) (based on specific filtration requirements, varying pore sizes, and separation efficiency) [12]. Membrane processes, according to driving forces, can be divided into equilibrium-based MPs, non-equilibrium-based MPs, pressure-driven MPs, and non-pressure-driven MPs [9,10].

Membranes based on synthetic organic polymers have been used particularly frequently for the separation of various substances (e.g., metal ions, pesticides, pharmaceuticals, synthetic dyes) from different solutions and in water purification processes due to their important properties, such as usually high mechanical, chemical, thermal, and corrosion resistance [13,14,15,16,17]. However, despite the dynamic development of the sector related to the design and production of new synthetic polymers, certain challenges related to, inter alia, the scarcity of petroleum resources, concerns about the environmental impact of the oil industry, and the cost of fossil fuels have led to numerous studies exploring the possibility of using biopolymers, which are renewable materials characterized by low toxicity and environmental friendliness for the production of membranes [15,17,18].

Although the group of naturally available biopolymers includes various substances (e.g., polysaccharides, polyphenols, polyamides), mainly polysaccharides, such as cellulose, chitin, starch, and alginate, were used (either as the main component or as filler) to produce membranes intended for the separation of various contaminants from effluents. Nevertheless, other than polysaccharide biopolymers, e.g., poly(lactic acid) or poly(butylene succinate) have also been successfully utilized for this purpose [17]. The field of designing new biopolymer-based membrane materials and applying them for the separation of various contaminants from aqueous solutions and suspensions is developing rapidly. In order to improve the performance of membranes, various modifications were introduced (e.g., coating, grafting, blending, introduction of additional components), which consequently led to the generation of a very diverse group of materials (in terms of composition and properties).

This paper presents an overview of the latest (mainly from the last two years) achievements in this field, with particular attention paid to the properties of novel membrane materials, their performance in the separation processes of dyes, salts, and metal ions from aqueous solutions and suspensions, and the potential impact of the developed MP-based separation methods on the natural environment.

## 2. Alginates

Alginic acid is a natural polysaccharide typically obtained from various aquatic organisms, e.g., kelp, seaweed, Ascophyllum, macroalgae, or by microbial fermentation with the participation of specialized bacteria. In organisms’ cells, it occurs mainly in the form of alginates. However, the chemical structure of both alginic acid and its salts is complex, and compounds originating from different species may differ, e.g., by polymer chain length and sequence of monosaccharides. In general, alginic acid consists of two conformational isomeric residues: β-D-mannuronic acid (M) and α-L-guluronic acid (G). Within the polymer, the M and G residues can be linked homogeneously (GG, MM) or heterogeneously (MG, GM) by 1–4 glycosidic bonds [19]. Thus, alginic acid and alginates constitute linear block polymers, and the ratio of G and M content (presence of GG, MM, and MG/GM blocks) influences the molecular structure of these compounds (e.g., the presence of GG units causes rigidity due to steric hindrance, the content of MM blocks increases flexibility, the most flexible structures contain many MG units) and, consequently, some of their properties. For example, at low pH, alginates with more MG/GM blocks are soluble, whereas those with more MM or GG blocks are less soluble or insoluble. Additionally, a large number of GG blocks in the polymer chain can affect the gelation process through the interaction with divalent metal ions (e.g., Ca^2+^) [19,20].

Alginates (e.g., sodium alginate), which, in addition to their exceptional gelling properties, are characterized by their renewable nature, biocompatibility, and biodegradability and can be relatively easily modified (e.g., through ionic crosslinking, plasticization and hybridization with other polymers and/or fillers), have found applications in numerous fields, such as the food, paper, and cosmetic industries [19,20].

In the case of many technical applications, their use could be limited due to poor mechanical properties and high susceptibility to water; therefore, the possibility of modifying them (e.g., through chemical derivatization, changes in the synthesis procedure, or the formation of (nano)composites) allows for giving such materials the desired properties [20,21,22]. Recently, alginate-based customized multiphase materials have played an important role in biomedical applications, as components for sensing, electronic and energy devices, and also in separation processes (e.g., in wastewater treatment) as components of adsorbents and membranes [20,21,22,23].

### 2.1. Hydrogel Alginate-Based Membranes

**Figure 1:**
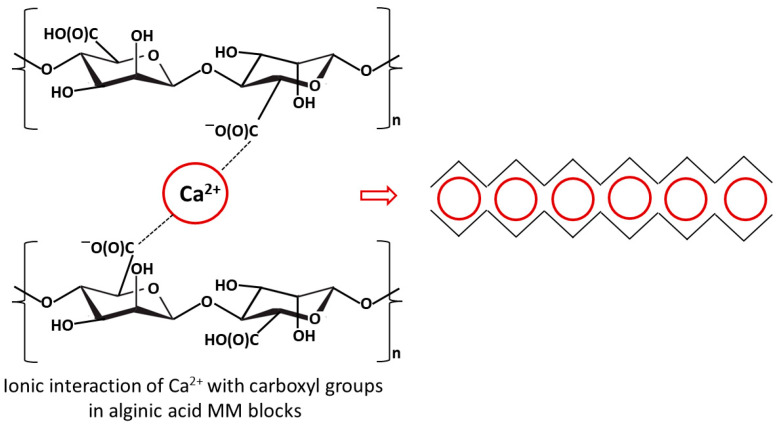
Simplified scheme of the ionic crosslinking mechanism of alginic acid (prepared on the basis of [19,20]).

In separation processes, among others, alginate hydrogels generated as a result of the interaction of G blocks present in different alginate chains and divalent cations, which leads to the formation of three-dimensional networks (the so-called “egg-box” model, in which divalent cations form bridges between guluronate blocks, enhancing mechanical stability and gel rigidity), were successfully used [24,25]. In the alginate gelation processes various metal ions can be utilized (e.g., Ca^2+^, Ba^2+^, Cu^2+^, Sr^2+^, Fe^2+^, Fe^3+^, Al^3+^), and some of them can also selectively bind to the carboxyl groups [26]. Figure 1 presents a simplified scheme of the ionic crosslinking mechanism of alginic acid.

Production of alginate hydrogels is relatively simple (e.g., alcohol-induced gelation followed by freeze-drying), and their effectiveness in removing various synthetic dyes and metal ions from aqueous solutions is usually high [24,25].

In relation to wastewater treatment processes, hydrogels have been divided into three main categories: hydrogel beads, hydrogel nanocomposites, and hydrogel membranes [12]. Of particular interest are hydrogel alginate-based membranes, in the case of which the appropriate selection of components allows to obtain materials free from typical membrane disadvantages, such as swelling, low mechanical durability, and easy contamination (organic and biological). For example, Gao et al. [27] prepared a co-crosslinked hydrogel alginate membrane Ba/CaAlg (by crosslinking sodium alginate with a blended aqueous solution of Ba^2+^/Ca^2+^) and applied it for the separation of Methyl blue and sodium chloride from dyeing wastewater. They showed that the Ba/CaAlg hydrogel membrane, compared to simple CaAlg material, exhibited a more stable structure (resistant to deformation), as well as salt tolerance. Moreover, the use of the Ba/CaAlg membrane for the separation of dyeing wastewater components enabled higher dye and lower salt rejection (>99.6% and <8.2%, respectively), and consequently, better separation of these substances.

Nowadays, however, alginate hydrogels are often used as components of more complex membranes. For example, it has been reported that a one-step ion-crosslinking method enabled the fabrication of a series of copper alginate (CuAlg) hydrogels, which assembled into a modified hydrophilic and mechanically robust microfiltration fiber support layer (MFSL) formed complex CuAlg/MFSL membranes that exhibited not only very good separation performance towards dyes and salts and high selectivity, but were also characterized by resistance to deformation, anti-swelling properties, antimicrobial capabilities, and worked well under high-salt conditions [28].

It should be emphasized that the development of novel membranes enabling the effective separation of main components from dye industry wastewater (i.e., dyes, metal ions, and salts) is crucial, both in terms of environmental protection and in relation to the possible recovery and reuse of these substances [29]. Separating dyes from such solutions is usually not easy, due to a large amount of salts, which should not be separated in the same process as the dyes (the dye separation process should be highly selective). Therefore, new, more effective, selective, and economical methods are being sought and, consequently, novel hydrogel membrane materials are being developed for this purpose. Table 1 shows selected examples of novel membranes produced using alginate hydrogels, utilized in the dye/salt/metal ion separation processes.

As can be concluded from the data presented in Table 1, one of the recent research directions in the development of new alginate-based hydrogel membranes enabling effective separation of dyes, salts, and metal ions from aqueous solutions (e.g., model solutions, wastewater) is the utilization for their generation of mainly sustainable materials such as, for example, chitosan (and its derivatives) and carrageenan [30,36]. Derivatives of naturally occurring chitosan (e.g., carboxylated chitosan, carboxymethyl chitosan) containing -NH_2_ and -COOH groups, whose presence can positively influence extensive hydrogen bonding and which may act as active binding sites for dye molecules, have been successfully used as components of different adsorption materials (e.g., composite adsorbents, electrostatically sprayed hydrogel microbeads), characterized by additional, desired properties (e.g., pH sensitivity, very high adsorption capacity) [40,41].

Carrageenan is a natural polymer derived from red marine algae, consisting of alternating D-galactose and 3,6-anhydro-D-galactose units linked via α-(1,3) and β-(1,4) glycosidic units. Carrageenan and its composites can be used to remove cationic dyes and various metal ions from aqueous solutions due to their reactive sulfate functional groups that facilitate adsorption through electrostatic interactions. As a component of alginate-based hydrogel membranes, it can also act as a crosslinkable agent, improving their mechanical properties by limiting swelling (anhydro-D-galactose units are less hydrophilic than the repeating disaccharide units of alginate) [36]. The interest in hydrogel membranes formulated using natural polymers or their derivatives is systematically growing, and such materials have even been called ‘green membranes’ due to, inter alia, the properties of their components (non-toxic, biodegradable) and the possibility of their generation by environmentally safe methods (no use of organic solvents, no high energy consumption) [30].

Another solution that fits into the research trend regarding the development of sustainable alginate-based hydrogel membranes is the use of cost-effective biochar for their production. For example, the application of biochar BCFS800 (ball-milled crayfish shell biochar with hierarchical pore structure, 289.7 m^2^/g surface area, abundant oxygen-containing groups), to formulate a composite hydrogel membrane based on alginate allowed for the formation of a uniform and dense gel layer on the matrix surface and increased efficiency of the membrane in the separation of small dye molecules and metal ions (nanofiltration membrane). Additionally, it has been reported that crosslinking by Ca^2+^ in biochar and alginate-containing membrane improved its mechanical stability [38].

Some of the recent research concerns the possibility of using the properties of graphene oxide to improve the mechanical strength as well as the anti-swelling ability of calcium alginate hydrogel membranes intended for efficient dye/salt separation. The two-dimensional nanostructured graphene oxide surface contains a large number of hydroxyl groups and abundant carboxyl groups at the sheet edges, which influence its adaptability to different materials. Graphene oxide-based materials used in separation processes possess unique nanoporous channels, which enable their application in, inter alia, dye desalination and metal ion separation. Hybrid hydrogel membranes containing alginate and GO have been shown not only to be suitable for efficient separation of Coomassie brilliant blue and NaCl but also to operate with good performance for a long time, and their utilization may provide a sustainable strategy for dye recycling [35]. Yu et al. [42] synthesized, using GO, a more complex membrane, which exhibited excellent dye separation performance, had great stability and, additionally, possessed the ability to “self-heal” after damage. A hydrogel-supported layered modified GO nanofiltration membrane was obtained in several steps (GO was grafted with ethylenediamine (EDA) to obtain EGO nanosheets, then oxidized sodium alginate (OSA) was introduced (the -CHO groups on the OSA molecular chain reacted with the active -NH_2_ groups, which led to the formation of a hydrogel network structure between adjacent GO layers through Schiff base reaction), and vacuum-assisted filtration has been utilized). “Self-healing” of the membrane after mechanical damage occurred after the addition of a small amount of OSA, due to the synergistic effect of the dynamic Schiff base and hydrogen bond interactions between OSA chains and EDA.

Generally, in the field of designing and developing new alginate-based hydrogel membranes, there is an increasing interest in the possibilities of giving them additional properties (besides satisfactory separation efficiency, mechanical strength, or swelling resistance), such as “self-healing” ability, reusability (after regeneration), or antibacterial properties [31,32,36].

In terms of antibacterial activity, the possibility of modifying gel membranes with nanoparticles (e.g., TiO_2_, silver nanoparticles (AgNPs)), which have strong antibacterial properties, seems to be particularly useful, especially with regard to the possibility of using such materials on a larger scale and for a longer period of time. Various nanoparticles have been used to modify both relatively simple membranes and complex composite membranes [31,32,43]. One of the limitations of their utilization may be the relatively poor dispersibility of nanoparticles in alginate gels, which needs to be improved so that such membranes can be used in the future in commercial applications. However, modifications were also introduced in this respect, e.g., Xu et al. [43] solved the problem related to the instability and agglomeration of silver nanoparticles by using bovine serum albumin (BSA), which has a strong affinity for inorganic nanoparticles. But because BSA-coated AgNPs have a weak interaction with alginate hydrogel and were easily washed out after longer storage, to overcome these limitations, it was necessary to introduce a larger number of components to produce a stable and effective VF-PET/BSA/CMCS/AgNPs/CaAlg (VF-PET—viscose fiber/polyethylene terephthalate fiber nonwoven fabric utilized as a support layer) membrane with antibacterial properties. The synthesized multi-component membrane was effective (removal of over 99.0% of Coomassie brilliant blue and less than 9.0% for sodium chloride), mechanically durable and has antibacterial properties (tested against *E. coli*).

Based on these examples, it can be concluded that determining the correct composition of membrane materials (qualitative and quantitative) is often not easy, but has a key impact on their properties and scope of application. To enhance the application potential of alginate-based hydrogel membranes, so that they can also be used for the removal of heavy metal ions, additional modifications/components were introduced to such materials. For example, it has been reported that the introduction of MOF and cellulose nanofibers to the hydrogel membrane enabled its use for efficient separation of dyes and heavy metal ions (Pb^2+^, Cu^2+^, Cd^2+^) [37]. NaAlg, CNF, PEG and micron-sized biochar (BC) containing membrane synthesized by in situ free water evaporation (“cooking”) process and ionic crosslinking were effective in the separation of Cr(III) and Cr(VI) ions from aqueous solutions (removal of 96.8% and 91.4%, respectively) [44].

Alginate hydrogels were also used in membranes intended for municipal sewage treatment for the removal of metal ions and in water desalination processes. For example, Wen et al. [45] applied Cu-alginate hydrogel to modify forward osmosis (FO) membrane (the FO process is driven by the osmotic pressure difference between the draw solution with higher solute concentrations and the feed solution with lower solute concentrations), which was successfully used to remove heavy metal ions (Cd^2+^, Cu^2+^, Pb^2+^) and for thickening of activated sludge produced during application of the popular activated sludge method in the wastewater treatment processes. They formulated a novel FO membrane (PES/Cu-SA/PA), through the formation of a polyamide (PA) selective layer on a Cu-alginate hydrogel (Cu-SA) intermediate layer, and modified polyethersulfone (PES) support (with the PA selective layer firmly “hooked” on the Cu-alginate intermediate layer due to the chelation crosslinking between the Cu^2+^ and –NH_2_ groups). They found that the hydrophilic trifunctional Cu-alginate intermediate layer enabled rejection of more than 96% of heavy metal ions (“creation” of a barrier for these ions due to the charge repulsion effect of the Cu^2+^ present in the membrane matrix) and effective dewatering and thickening of activated sludge. Both achievements are important because heavy metal ions are among the main contaminants in wastewater and can pose a serious threat to living organisms, whereas activated sludge containing more than 99% moisture can only be further processed (incinerated, composted) after the excess water is removed.

Amiri et al. [46] reported that alginate hydrogel can be utilized as a component of the more complex membrane PVA-NaAlg-GO (prepared through the surface coating and in situ crosslinking procedure, containing polyvinyl alcohol and sodium alginate as coatings, graphene oxide as a hydrophilic additive, polysulfone as a support and glutaraldehyde as a chemical crosslinking agent), effective both, in the process of removing hazardous As(III) ions (removal of 88.4%) and in the desalination processes (rejection of 60% of NaCl, 74.5% of MgSO_4_, and 92.0% of Na_2_SO_4_). The membrane was also characterized by good fouling resistance, which is important in relation to the possibility of its use for a longer time.

### 2.2. Aerogel Alginate-Based Membranes

Alginates can also be used to produce aerogels, utilized in different fields, which, like hydrogels, usually must be modified to provide greater mechanical strength, for example, by introducing strong covalent crosslinkers or stiff reinforcements. Various substances can be used as these additional components; some of them may not be eco-friendly [22]. When developing new alginate-based aerogel membranes intended for the separation of dyes or metal ions, not only are the properties of the components (and their mutual influence) of the designed material taken into account, but attention is also paid to ensuring that the synthesis process is not overly complicated. For example, Liu et al. [47] obtained a strong, triply crosslinked alginate/graphene aerogel using economical precursors and a simple synthetic process. The triple crosslinking of aerogel was achieved by the association between in situ released Ca^2+^ ions and sodium alginate, electrostatic attraction between polyethyleneimine-modified reduced graphene oxide (PEI-RGO) and the NaAlg chain, and by the hydrogen bonds between PEI and alginate. They found that the addition of PEI-RGO improved the mechanical properties of the material, modifying the porous structure and increasing the number of active sites. Such material, used to remove cationic dyes (Methylene blue, Neutral red) from wastewater, allowed for their effective separation (removal of 92.2% and 91.9%, respectively, in optimal experimental conditions), which suggests its potential application in sewage treatment.

In the case of wastewater treatment, the development of separation materials that can simultaneously remove complex contaminants (e.g., heavy metals, organic dyes, microorganisms, and oil contaminants) that typically coexist is of significant practical importance. Wang et al. [48] reported that calcium alginate aerogel membrane with well-selected components can be successfully used for this purpose. They prepared a novel aerogel membrane by ice spraying, polymerization, Ca^2+^ crosslinking, and freeze-drying using aniline and sodium alginate as raw materials without the utilization of environmentally harmful raw materials. The obtained membrane enabled separation of oil–water emulsion under gravity with very high efficiency (99%), and at the same time, it effectively adsorbed organic dyes (removal rates of Methylene blue and Congo red of about 92% and 63%, respectively) and heavy metal ions (95% of Pb^2+^). Additionally, the synthesized membrane showed photothermal conversion ability and antibacterial ability (analyzed towards Staphylococcus aureus) and was characterized by low cost, simple preparation, good stability, and high recycling ability. All these advantages mean that this aerogel membrane has potential application prospects in wastewater treatment.

Another material efficiently used to simultaneously remove oil, dyes, and bacteria from aqueous solution was an aerogel calcium alginate-based membrane (CTW) prepared through sol spraying, Ca^2+^ crosslinking and freeze-drying by utilizing tetrabutylammonium hydroxide (TBA) quaternary ammonium-salt-modified sodium alginate as raw material and waterborne polyurethane (WPU) as adhesive. Due to its super-hydrophilic and underwater super-oleophobic properties, the CTW enabled efficient one-step separation of oil–water emulsions under gravity (>99%) and removal of bacteria (100%), as well as Congo red dye (99%) from aqueous solution. Additionally, a significant advantage of this membrane, both economically and environmentally, is its recyclability [49].

The numerous advantages of alginate-based aerogel membranes, e.g., high strength, high efficiency in separating dyes, metal ions, and oils, the possibility of using various components for their formation, including combinations of different biodegradable biopolymers to give them specific properties (“tailor-made”), and the possibility of their multiple use, make these materials promising in the field of wastewater treatment [50].

The examples of novel membranes described in this chapter reflect attempts to reduce the main disadvantages of alginate-based materials, which include their not-always satisfactory mechanical stability, often insufficient separation efficiency, their susceptibility to fouling, and the possibility of microorganism growth on the membranes’ surfaces. It has been shown that the low mechanical stability of such materials (associated with low internal strength and a tendency to swell), can be significantly improved by, for example, crosslinking (e.g., with Ba^2+^ and Ca^2+^ ions), the use of environmentally friendly nonwovens, or the introduction of multifunctional additives (e.g., carrageenan, which “provides” additional groups enabling the binding of dyes and participates in crosslinking) [32,36]. Improvement in the selectivity and separation properties of alginate-based membranes can be achieved by introducing various substances containing numerous functional groups [34,35]. The addition of nanoparticles (e.g., TiO_2_, silver nanoparticles) improves the antibacterial properties of alginate-based membranes. However, the synthesis of such complex materials typically requires additional steps (e.g., usually due to the poor dispersibility of nanoparticles in alginates) and is associated with the use of additional reagents and lengthens the membranes’ preparation process [42]. A certain limitation, in terms of the potential use of alginate-based membranes for the separation of dyes and metal ions from aqueous solutions and suspensions, is also the relatively poor data on the possibility of their repeated, long-term utilization and their environmentally safe disposal after use. Furthermore, some recent studies have focused on the use of new membranes in simple model solutions, and it is unclear how these materials will perform in more complex, real wastewater applications.

## 3. Cellulose and Its Derivatives

Cellulose, a polysaccharide biopolymer in which D-glucose units are linked by β-1,4-glycosidic bonds, is not only one of the most common biomass resources, but it is also a promising material utilized in various fields, including the production of different types of membranes. In general, cellulose exhibits anisotropic mechanical properties (mechanical behavior changes depending on the direction of the applied force), but novel cellulose materials usually resist deformation under the influence of external forces. Furthermore, they have a relatively low density, which contributes to their light weight. The properties of natural cellulose, such as the possibility of the formation of intramolecular and intermolecular hydrogen bonds (due to the content of abundant hydroxyl groups) and hydrophobic interactions (thanks to its carbon skeleton), influence its insolubility in many solvents, which is important in terms of potential utilization of this material in the formation of various types of solvent resistant membranes. Cellulose-based membranes can also be formed by using its solutions obtained by utilization of special solvents (e.g., *N*-methylmorpholine oxide, ionic liquids), which expands the possibilities for producing such materials [51,52]. Other important properties of cellulose include degradability, biocompatibility, and the possibility of relatively easy modifications of its surface.

Cellulose can occur in various forms, as the extensive hydrogen bond network created due to the presence of hydroxyl groups gives this substance a variety of partially crystalline fibrous structures and morphologies. To produce separation membranes, among others, plant-derived cellulose (e.g., obtained by acid/alkali treatment and oxidation processes of celluloses originating from wood and cotton), bacterial cellulose (synthesized by bacteria), nanocellulose (cellulose materials with at least one dimension in the nanoscale range, e.g., cellulose nanofibers and cellulose nanocrystals) and various cellulose derivatives can be used [53]. However, despite the possibility of using materials originating from various sources and of different natures (e.g., natural and derivatized cellulose) to produce cellulose-based membranes and applying them to separate various pollutants, their potential has not yet been fully exploited. Challenges associated with manufacturing and utilizing cellulose membranes on a larger scale (especially in the case of those directly fabricated from cellulosic materials) include, inter alia, the need to improve their efficiency and to limit energy consumption during raw material preparation, the problems with selection of the appropriate green and efficient solvents suitable for membrane formation, the typically short membrane lifespan, and the high overall costs [53]. An important problem is also the tendency of cellulosic materials to absorb water, which can affect their mechanical properties [52].

In recent years various cellulose derivatives (e.g., cellulose acetate (CA), carboxymethyl cellulose (CMC), cellulose acetate butyrate (CAB), cellulose acetate propionate (CAP) and methyl cellulose (MC)) have gained particular interest, as their utilization in membranes, including complex membranes containing several components, enables improvement in membranes’ specific properties and their performance [52,53,54,55]. Table 2 presents selected examples of the novel cellulose and cellulose derivative-based membranes intended for the separation of dyes, salts, and metal ions, reflecting the latest research directions in the development of new separation materials.

**Figure 2:**
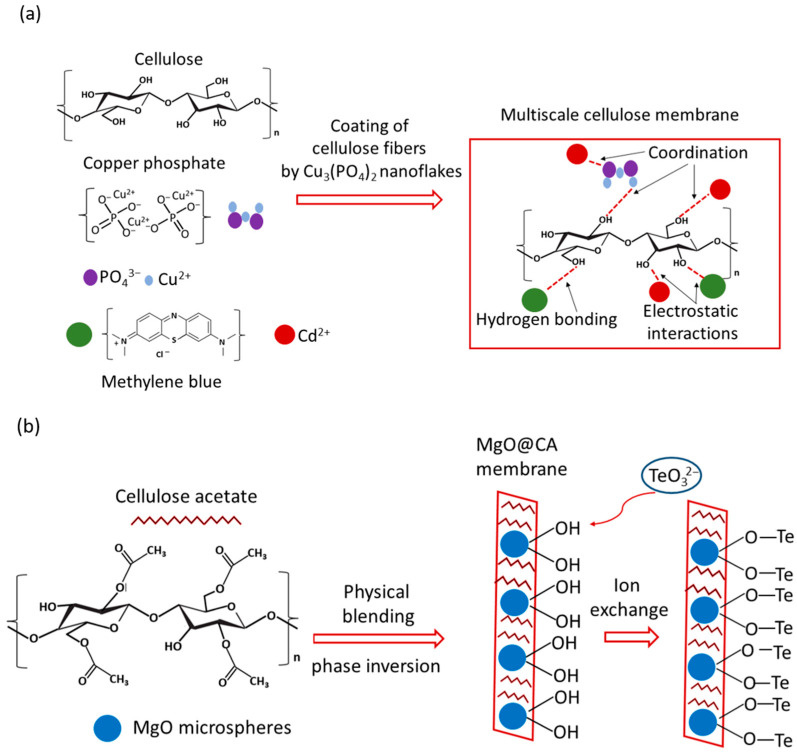
Simplified scheme of possible separation mechanisms of dyes and metal ions using cellulose-based membranes; (**a**) the multiscale cellulose membrane (MCM) prepared from crop straw and coated with functional copper phosphate (CP) nanoflakes intended for separation of Methylene blue and cadmium ions. (**b**) Porous magnesium oxide-modified cellulose acetate membrane (MgO@CAM) prepared via a physical blending and phase inversion method intended for tellurium ions separation (prepared on the basis of [68,69]).

Recent research directions in the generation of cellulose-based membranes have been related to the utilization of this substance in different forms (e.g., cellulose nanocrystals, cellulose nanofibers—pristine and functionalized) and originated from various sources (e.g., wood cellulose, bacterial cellulose) [56,57,58,64]. Additionally, the possibility of using a variety of cellulose derivatives and additional substances for separation material synthesis has led to the formation of a wide range of membranes with diverse properties. The mechanisms of the separation of dyes and metal ions by such membranes depend, inter alia, on the composition of these novel materials and may vary. Figure 2 shows a simplified scheme of possible separation mechanisms for two different types of cellulose membranes.

Among the novel separation materials, 2D layered membranes characterized by high permeability and selectivity are of particular interest. In general, they are produced through self-assembly and stacking of 2D nanomaterials (e.g., graphene-based materials, covalent and metal–organic frameworks, and MXene nanosheets), which exhibit layered structures at the atomic level, and are capable of forming thin separation layers with regular slits between the layers [57,60,62]. However, these “additional” substances differ in their properties, and membranes containing them are often enriched with other components to impart the material’s desired properties. Such membranes often have a complex composition and their synthesis is multi-stage, but the obtained composites are multifunctional.

For example, it has been reported [63] that GO, which has abundant oxygen-containing groups (they enhance the hydrophilicity of the membrane), a high specific surface area, and a 2D lamellar structure, can be used to fabricate two-dimensional lamellar cellulose containing membranes. However, the use of GO alone does not solve the problems of membrane fouling and bacterial growth. Novel membranes characterized by the appropriate efficiency, selectivity (towards dyes), mechanical strength, resistance to fouling, and antimicrobial properties have been formulated in a several-stage procedure with the utilization of a variety of substances. First, ethylenediamine has been introduced into the GO structure via high-temperature grafting to formulate functionalized nanosheets (EGO); then, silver nanoparticles were formed in situ on dialdehyde cellulose nanocrystal (DACNC) molecular chains and, finally, a crosslinked network has been constructed by the Schiff base reaction between DACNC-AgNPs and EGO nanosheets. Dialdehyde cellulose nanocrystals were utilized instead of unmodified cellulose because aldehyde groups are more reducible than hydroxyl and carboxyl groups and are able to reduce Ag^+^ to silver nanoparticles (which have the ability to enrich silver in situ and have optical, catalytic, and antimicrobial properties) more efficiently. This new material offers additional advantages over traditional cellulose-based membranes, including photocatalytic, antibacterial, and antifouling properties and an extended service life [63].

When designing new composite membranes, the ability to develop relatively uncomplicated and fast methods for their cleaning (e.g., “self-cleaning”), which enables their repeated use, is also important, both in terms of environmental protection and economic issues. For example, intensive research was underway on the techniques enabling fast and direct degradation of contaminants (e.g., dyes) separated from aqueous solutions [57,58,61,63]. It can be assumed that multifunctional composite membranes will play an increasingly important role in dye wastewater treatment processes. However, it should be emphasized that various cellulose derivatives are increasingly tested for possible applications in novel membrane formation, and one of the important research directions is also the development of simple and economically feasible methods for preparing materials enabling efficient dye/salt separation [68,70]. An important research direction in the production of membranes based on cellulose or its derivatives is also the development of green methods for the synthesis of these materials, which involves the use of environmentally sustainable and low-toxic alternatives (e.g., solvents) [71,72,73].

### Cellulose Triacetate-Based Membranes

Among the numerous cellulose derivatives, cellulose triacetate plays an important role in the production of various types of membranes. Due to its properties (e.g., resistance to bases and acids, good solubility in various organic solvents, thermal and physical stability, and relatively low price), this compound is particularly frequently used as a matrix in polymer inclusion membranes (PIMs) intended for separation of various metal ions (hazardous heavy metal ions, valuable precious metals, and rare earth metal ions). PIMs, in which the liquid phase is held within the network of a polymer, also contain various substances as carriers (responsible for binding metal ions from aqueous solutions and for their transport into the membrane structure) and, usually, a plasticizer (which influences the physical and chemical properties of the membranes). Polymer inclusion membranes are characterized by many advantages such as, among others, the ease of modifying their composition (qualitatively and quantitatively, “tailor-made” membranes), relatively simple formation methods, the possibility of reducing the consumption of hazardous organic solvents (during their production and use), the possibility of conducting simultaneous extraction and back extraction, good stability and high reusability, and the simplicity and relatively low costs of the membrane processes. The disadvantages of PIMs include efficiency and selectivity that are not always sufficient, with respect to specific metal ions or susceptibility to fouling [74,75].

Hence, new PIMs are systematically developed, including those based on CTA, which perform better under specific conditions, such as various types of wastewater characterized by different pH values and containing different contaminants, including those other than the removed/recovered metal ions. For example, Chaijan et al. [76] reported that CTA(matrix)/TOA(carrier)/TBP(plasticizer) PIM can be successfully used for efficient and selective extraction of cobalt(II) ions from thiocyanate-containing leachates of Ni-Cd batteries. Additionally, they found that cobalt(II) ions were extracted as their anionic thiocyanato-complexes via the formation of [TOAH^+^]_2_[Co(SCN)^2−^] ion pairs. Since cobalt plays a significant role in many industries, but its excessive entry into the human body seriously threatens health, it is necessary to develop effective methods for removing cobalt ions from aqueous solutions, such as industrial wastewater or waste leachates. Earlier Ghaderi et al. [77] applied PIM of similar composition to separate bismuth (III) ions from a multi-component solution and reported that the membrane showed excellent selectivity towards Bi(III) over Cu(II), Pb(II), Zn(II), Ni(II), Co(II), Cd(II), Fe(III), Cr(III), Mo(VI), W(VI), NO_3_^−^, and SO_4_^2−^ in various aqueous solutions (samples of spiked bismuth(III) well water, tap water, seawater, a leach soil sample from a zinc production plant). Additionally, they showed that the PIM-based separation strongly depended on the process conditions (e.g., the low extraction percentage of bismuth from the leach solution was related to the high concentration of sulfate and a possible competition of these ions), and the membrane after regeneration can be used several times (in four subsequent extraction/back-extraction cycles). Moreover, they also found that the replacement of TBP plasticizer by other substances, such as 2-nitrophenyloctyl ether or dibutyl phthalate (DBP), increased the stability of the formed PIMs. The demand for low-toxicity bismuth stems from its widespread use in various sources (e.g., pharmaceutical, cosmetics, chemical, and metallurgical industries). Hence, there is continued interest in new, efficient methods and processes for separating and recovering bismuth from various sources.

More complex membranes containing a combination of biopolymers (or their derivatives, such as CTA) and synthetic polymers are also being developed more and more often. Recently, Safari et al. [66] introduced another modification to the CTA/TOA/TBP membrane, using a blend of CTA and poly(vinylidene fluoride-co-hexafluoropropylene) (PVDF-HFP) polymers as a matrix. They demonstrated that the blend polymer inclusion membrane BPIM (CTA/PVDF-HFP/TOA/TBP), with appropriate selection of membrane components and membrane process conditions, is suitable for effective separation of bismuth (III) ions from the residue of a zinc plant production. These examples of membrane material modifications confirm that changing the membrane’s components can significantly impact the efficiency of an MP under specific experimental conditions.

Membranes with well-known composition were also modified to be useful in new experimental conditions to expand their applications. One of the research directions in the development of new CTA-based polymer inclusion membrane materials intended for the selective separation of specific metal ions is the utilization of combinations of chemical compounds known for their metal ion binding properties as ion carriers. For example, Ntombela et al. [78] synthesized novel polymer inclusion membranes with CTA as a matrix, a mixture of dibenzylmethane (DBM) and trioctylphosphine oxide (TOPO) as carriers (in different weight ratios), and 2NPOE as a plasticizer, and used them for lithium extraction from model solutions and seawater containing other metal ions (e.g., Mg(II), Ca(II) and Na(I) ions in high concentrations) and reported that, under appropriately selected experimental conditions (e.g., membrane composition, pH and composition of feed and receiving solutions), the PIMs enabled the selective separation of Li(I) ions. Whereby, slightly changing the membrane composition with respect to the carriers (60 wt% of DBM/TOPO in a (1:1) or (2:1) ratio) influenced the order of extraction (Li^+^ > Na^+^ > Ca^2+ ^> Mg^2+^ and Li^+^ > Ca^2+^ > Na^+^ > Mg^2+^, respectively). Shen et al. [79] fabricated a CTA-based PIM with TBP along with sodium tetraphenylborate (NaBPh_4_) as carriers and used such membranes for selective and permeable Li(I) ion separation from high-Mg(II)/Li(I)-ion aqueous solutions. They reported that PIM performance was influenced by the concentration of carriers and other membrane components. Moreover, the moderate TBP content in the membrane could enlarge the distance of CTA chains and enhance the initial flux of PIMs, whereas excessive TBP can occupy the free volume sites, which adversely affects permeability. The obtained results may be important in the future development of methods for lithium extraction from seawater, which is still a challenge due to the presence, in it, of several other metal ions in very high concentrations (especially compared to lithium ion concentration).

Lithium is considered a crucial element due to its wide diverse applications in key industries, and seawater may be treated as a valuable reservoir of this raw material [80]. CTA-based PIMs containing various carriers and plasticizers have also been successfully utilized for efficient separation and recovery of other valuable raw materials from aqueous solutions (e.g., WEEE leachates, wastewater), such as precious metal ions (e.g., gold, silver, platinum) [81,82,83].

Recently, research has also been conducted on the possibility of wider use of CTA-based PIMs, e.g., for the formation of passive samplers intended for metal ion monitoring in rivers and for studying metal–contaminant interactions in aquatic environments. For example, Alcalde et al. [84] used PIM-based passive sampler (PIM composed of CTA(matrix)/D2EHPA(carrier)/2NPOE(plasticizer)) to monitor a mine-contaminated river (in situ sampling) in relation to zinc ion contamination and reported that the membrane allowed the transport and preconcentration of free Zn species from the sampling medium to the receiving phase (HNO_3_ solution). Passive sampling based on the free flow of the analyte from the sampled medium to a receiving phase of the passive sampling device, as a result of the difference in chemical potentials, has been widely used for the monitoring of various pollutants in natural waters because this technique enables simultaneous collection, separation, and preconcentration of specific analytes in the receiving phase. However, in the case of monitoring hazardous metal ions in waters by passive sampling, mainly methods based on diffusive gradient in thin film (DGT) or Chemcatcher samplers were used. The application of CTA-based PIMs for the control of Zn ions in river waters is innovative, although the possibility of using various composition PIMs for metal ion monitoring has also been considered earlier [85,86,87]. The obtained results indicate that PIM(CTA)-based passive samplers can potentially be a valuable tool for environmental monitoring in the future.

Khatir et al. [88] applied a novel approach based on the utilization of PIM (CTA/D2EHPA/TBP)-based separation for studying divalent metal ion (Ni(II), Cu(II), and Zn(II)) interactions with sulfonamide antibiotics and two types of microplastics (commercial PVC and a mixture of plastic waste) in aquatic environments. Because the application of PIM enabled the selective extraction of metal ions, the free fraction of metals could be easily assessed, which in turn was important for understanding their bioavailability and potential toxicity. The authors demonstrated that PIM effectively facilitated the transport of Ni(II), Cu(II), and Zn(II) into a 0.5 M HNO_3_ receiving phase, with an affinity following the trend of Cu(II) > Zn(II) > Ni(II). All metals exhibited a linear trend in relation to their concentration in the feed phase and their amount accumulated in the receiving phase. The obtained results showed that the analyzed metal ions did not interact with examined contaminants. Furthermore, the synthesized PIM was successfully used to measure free zinc ion levels in river water samples contaminated by drainage from an abandoned mine, as well as in the same matrix enriched with microplastics and antibiotics at environmentally relevant concentrations. Investigating the extraction of divalent metal ions in the presence of pollutants typical for many water reservoirs provides insight into real conditions and allows for a better understanding of the reactions that may occur in the environment.

In summary, interest in new membrane materials based on cellulose and its derivatives (e.g., CTA) is steadily growing, and the scope of applications for such membranes is expanding (separation of specific contaminants, formation of passive samplers, studies of interactions between various contaminants) [84,88]. Research is also being conducted on the possibility of using other materials based on cellulose derivatives, e.g., cellophane films and on the utilization of recycled cellulose (e.g., cellulose acetate (CA) derived from discarded cigarette butts) [89,90]. Recycled cellulose has been used for the formation of complex membranes intended for lithium ion separation; such a solution is associated with fostering sustainable and environmentally responsible innovations [90].

Despite the great interest in membranes based on cellulose and its derivatives, their production and use for the separation of dyes and metal ions from wastewater (especially on a larger scale) is limited by, among others, the costs of obtaining the necessary raw materials and the relatively limited possibilities of using environmentally safe solvents during their production [53]. Moreover, more complex cellulose-based membranes characterized by high efficiency and adequate stability usually contain additional substances, including environmentally non-neutral chemical compounds or synthetic polymers [59,62,64,66], which means that such materials cannot be fully considered biodegradable and eco-safe. In order to confirm the “environmental friendliness” of such materials, it is necessary to carry out in the future appropriate tests in this area (e.g., regarding their toxicity, mechanisms and products of degradation after use, etc.).

## 4. Starch and Cyclodextrin

### 4.1. Starch-Based Membranes

Starch, a polymeric carbohydrate with numerous glucose units linked by glycosidic bonds, consists of two types of alpha-glucan, amylose (linear), and amylopectin (branched), occurring in varying ratios, which depends on its botanical origin. It is a promising, sustainable material due to its wide availability, low cost, and ease of modification (e.g., thanks to the abundance of hydroxyl groups in the main chain). Starch can be simply modified, e.g., by acylation, esterification, oxidation, crosslinking, and grafting, and customized starch with desired properties has recently been used in different dye separation processes, e.g., flocculation and adsorption [91,92,93]. Both starch and its derivatives were also successfully utilized in the formation of various types of materials (membranes, nanocomposites, hydrogels) intended for the effective removal of dyes and metal ions from aqueous solutions [94,95]. However, starch-based separation materials have certain limitations, e.g., many of them may effectively remove only certain dyes and metal ions, they are not always selective, and their regeneration and reuse may also pose significant challenges. To address these limitations, chemical modifications of starch and/or additional substances (e.g., crosslinking agents) are usually introduced to increase such materials’ separation capacity and selectivity, as well as to optimize their structural integrity. However, modifications may also be associated with complicating the synthesis and increasing separation process costs [95]. Therefore, in order to obtain separation materials, such as membranes, characterized by desired properties and satisfactory performance in the removal of dyes and metal ions, new solutions are being sought.

The production of new starch-based membranes usually requires the use of several components, including synthetic polymers and multi-stage synthesis, and is associated with the need to determine the optimal conditions for separation processes (both in terms of the membrane composition and the separation process itself). For example, Ghanbari et al. [96] used corn starch to fabricate nano-starch incorporated PVDF mixed matrix membranes intended for the separation of various types of dyes. They used a one-pot, simple strategy for the synthesis of core-shell nano-starch, in which starch aldehyde produced by the oxidation of corn starch was subjected to a mercaptoacetic acid-locking imine reaction (MALI reaction, a type of green, catalyst-free CLICK reaction occurring at room temperature with small amounts of by-products). Next, they used polyethyleneimine (PEI), which has antibacterial activity and positive surface charges, to synthesize the core-shell structure (a strong interaction between the negatively charged core and the shell). The resulting core-shell antibacterial nano-starch nanoparticles consisted of numerous hydrophilic groups, which influence the membrane’s antifouling properties, and were incorporated into the polyvinylidene fluoride membrane matrix. Although starch itself is characterized by poor miscibility with other polymers and can be easily contaminated with bacteria, the use of a number of other substances (e.g., dopamine, thioglycolic acid, PEI) and an appropriate synthesis method to produce the membranes allowed for reducing these limitations. Moreover, the optimized antibacterial membrane enabled not only very efficient separation of various dyes (removal of 99.8%, 99.1%, 99.6%, 95.8%, and 97.9% of Methyl green, Methylene blue, Crystal violet, Reactive red 120, and Direct yellow, respectively) but was also characterized by long-term stability.

Huang et al. [97] formulated novel membranes by coating PVDF with dialdehyde-soluble starch (DAS) and PEI, to improve such separation materials’ antifouling and multifunctional properties, and utilizing a combination of dip-coating and spray-coating techniques. They produced two types of membranes, one in which DAS was used as the outermost layer (the PVDF membrane was immersed into PEI solution and, then, the DAS solution was sprayed onto the membrane surface) and the other with PEI as the outermost layer (the PVDF membrane was immersed into DAS solution, and then the PEI solution was sprayed onto its surface). The positive charge of PEI and the negative charge of DAS contributed to the high separation efficiency of membranes with optimized composition and removal of 99.1% of anionic dye Methyl orange (with PVDF/DAS/PEI) and of 88.3% of cationic dye Rhodamine B (with PVDF/PEI/DAS). In addition, the authors reported that the coating layer was stable due to the crosslinking of DAS and PEI.

These examples are in line with current research directions regarding the development of environmentally friendly separation materials, including bio-nano-hybrid composite membranes based on polysaccharides, their derivatives, and water-soluble polymers, offering innovative solutions for counteracting environmental pollution with dyes. Another example of new “green” materials that fit into this trend are those developed by Illas et al. [98], who formulated starch/chitosan nanoparticle bio-nanocomposite membranes by incorporating, into starch biopolymer, different concentrations of chitosan nanoparticles (CNP, fabricated by the ionic gelation method). The optimum starch/CNP(10%) membrane exhibited a smooth surface and high porosity and enabled the efficient removal of Methylene blue (94.0%) after the filtration.

In the case of membranes developed in recent years, intended for the removal of heavy metal ions from aqueous solutions, starch was mainly used as one of the components of multi-component separation materials. For example, Azzam et al. [99] formulated composite ceramic membranes (CCM) using available and cheap raw substances as a ceramic supporting filter (i.e., ball clay, kaolin, feldspar, quartz), corn starch flour as a pore-developing agent, and a polyamide 6 thin layer as a coating, improving these new materials’ separation performance. Such ceramic composite membranes have been used for removing several metal ions, including heavy metal ions (e.g., Li(I), Cu(II), Cd(II), Fe(III), Mn(II), Ni(II), Sb(III), Cr(III), Zn(II), Co(II), and Al(III) ions) from agricultural wastewater. It has been reported that the membranes enabled effective separation of metal ions (from over 80% to 99.97%, depending on the membrane composition and utilized fabrication method (e.g., sintering temperature, soaking time)) from complex agricultural wastewater. Importantly, the produced membranes were inexpensive, easy to prepare using locally available and cheap materials, could be reused, and were characterized by limited fouling. All these advantages allow for the assumption that membranes of this type can potentially be used on a larger scale in the future to remove heavy metal ions from agricultural wastewater.

It has been shown that starch is also useful for the production of membranes intended not only for the separation of metal ions from aqueous solutions but also in research related to ion speciation, which is important, for example, in relation to water monitoring (e.g., precise determination of Cr(III)/Cr(VI); Cr(VI) is highly toxic). Recently, Vasileva and Karadjova [100] synthesized a novel hybrid hydrogel nanocomposite membrane which can act as an effective sorbent for the simultaneous speciation and determination of the valence species of chromium and manganese in water samples using selective solid-phase extraction. They used poly(vinyl alcohol) and poly(ethylene oxide) as film-forming compounds and porogen agents, and silica sol and starch-coated gold nanoparticles as crosslinking and mechanically stabilizing components to formulate PVA/PEO/SiO_2_/AuNPs(starch) membranes. They reported that, under optimal experimental conditions, Cr(III) and Mn(II) could be simultaneously adsorbed onto the formulated membrane (retained on the negatively charged membrane), while Cr(VI) and Mn(VII) remained in solution (due to electrostatic repulsion). The proposed method based on the PVA/PEO/SiO_2_/AuNP(starch) membrane is simple, and detection limits as well as analytical precision make it potentially suitable for monitoring water quality standards.

The versatility of starch, in terms of its possible use in various separation materials, leads to its increasingly broader applications, for example, as a sustainable material intended to reduce metal ion surface contamination. Recently, Anggakusuma et al. [101] formulated a cassava starch–glycerol–water mixture and applied the resulting gel to glass, aluminum plates, and ceramics contaminated with heavy-metal stable ions corresponding to a radionuclide. They reported that starch–glycerol gel was effective in metal ion removal (high adsorption related to many -OH groups in the polymer) and forms an easily peeled-off (after drying) film. The obtained results suggest that the starch–glycerol gel can be used as an alternative method for decontamination of various material surfaces.

In summary, starch, offering advantages such as hydrophilicity, biocompatibility, and ease of chemical modification, has been used over the past decade to fabricate membranes that have demonstrated effectiveness in micro-, ultra-, and nanofiltration for various contaminant removals. Challenges associated with using native starch (e.g., its sensitivity to water and limited mechanical strength) have been addressed through chemical modifications, polymer blending, nanoparticle incorporation, and crosslinking, which improved membrane stability and selectivity. Advanced fabrication methods have also improved membrane properties and performance. It can be assumed that future research in the utilization of starch for separation membrane production will primarily focus on advanced starch modification strategies, the development of composite systems with novel nanomaterials, and eco-friendly synthesis methods [102].

### 4.2. Cyclodextrins-Based Membranes

**Figure 3:**
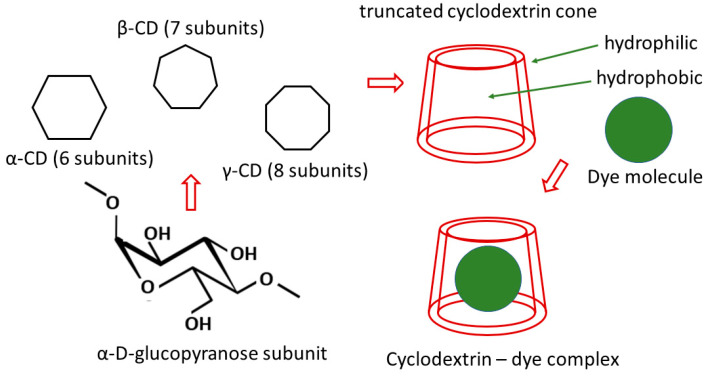
Simplified scheme of the formation of a cyclodextrin-dye molecule complex (prepared on the basis of [103,104,105]).

Cyclodextrins (CDs), natural polysaccharides produced by bacterial degradation of starch, consisting of a series of α-D-glucopyranose subunits joined by α-1,4-glycosidic bonds, have been divided due to differences in their structures into α-CD, β-CD, and γ-CD, which contain six, seven, and eight d-glucose subunits, respectively. The chair conformation of the d-glucose subunit causes the three native cyclodextrins to form a “truncated” cone, which influences the free hydroxyl groups’ orientation. The hydroxyl groups point towards the outside of the CD molecule, with the C2 and C3–OH groups pointing towards the wide rim and the C6–OH groups pointing towards the narrow rim. The exterior of CDs is polar and the interior cavity is hydrophobic (it is “lined” with non-polar groups). The hydrophobic cavity has the ability to encapsulate small organic molecules, which has been used in many fields and has led to the wide use of cyclodextrins, e.g., in medicine and in the food industry [103]. The properties of cyclodextrins, such as non-toxicity, stability in water and some organic solvents, good compatibility with polymers, and the ability to form stable inclusion complexes with guest molecules (GM) via various mechanisms (e.g., van der Waals forces and hydrogen bonding interactions between CDs and GM and polar–polar interaction between CDs and GM), have led to their use for the formation of different separation materials intended for the removal of dyes and metal ions, including membranes [104,105]. Figure 3 shows a simplified scheme of cyclodextrin inclusion complex formation.

Cyclodextrins can be used to produce various membranes with diverse properties. Recently, membranes produced by interfacial polymerization (which involves dissolving monomers in two immiscible phases and then polymerizing at their interface) have gained significant interest. As a result of the utilization of interfacial polymerization, cyclodextrins can be arranged in an orderly manner to form a membrane with well-defined sub-nanometer channels. Such membranes are usually characterized by high efficiency with respect to various substances and their synthesis is usually easy and quick [106]. For example, Zhou et al. [107] used the interfacial polymerization method to synthesize new monomer cyclodextrin-pentaethylenehexamine (CD-PEHA) containing a CD cavity and long amino chains as a building block to fabricate CD-embedded polyamide nanofilms intended for the separation of lithium (I) and magnesium(II) ions. The development of efficient, cheap, and environmentally safe methods for the separation of lithium and magnesium ions is important in the recovery of valuable Li(I) (e.g., from brines, waste leachates), because these ions usually occur simultaneously. It has been reported that in the case of CD-PEHA, protonated amino groups and the shape of the CD cavity intensified the free volume and electropositivity of the membrane, which was favorable for Mg(II)/Li(I) ion separation.

However, not only cyclodextrins but also their derivatives were successfully used to produce various membranes intended for removing dyes and metal ions from aqueous solutions. For example, Łagiewka et al. [108] synthesized, through a simple solvent casting method, a novel PIM containing perbenzylated β-cyclodextrin derivative as the carrier, CTA as a matrix, and o-NPOE as a plasticizer and used such materials for the separation of two different types of dyes, cationic Methylene blue and anionic Acid Orange 7. They reported that membranes containing cost-effective carriers enabled the effective removal of dyes with strong selective performance, depending on the conditions of transport experiments and the chemical nature of the removed dyes. The analysis of the molecular mechanism of the removal process led to the conclusion that it was mostly based on the complex inclusion of dyes inside the CD cavity.

Recently, Gao et al. [109] formulated a novel nanofiltration membrane intended for the separation of Li(I) and Mg(II) ions by combining quaternized γ-cyclodextrin (Q-γ-CD) with a PEI aqueous solution and forming of a thin film composite (TFC) on the surface of a homemade PVDF. Utilization of Q-γ-CD containing positively charged quaternary ammonium groups on its surface enhanced the positive charge density of the formulated membrane surface (it improved the rejection of divalent cations, e.g., Mg(II)), while the size and hydrophobic inner of the Q-γ-CD cavity enabled rapid transport pathways for lithium ions and water molecules. Novel, Q-γ-CD-based, positively charged nanofiltration membranes enabled the achievement of efficient magnesium–lithium separation with the rejection of 97.8% of magnesium ions.

Cyclodextrins have also been used to fabricate more complex loose nanofiltration membranes. For example, Guo et al. [110] immobilized β-CD within the pores of the substrate membrane via thermal shrinkage of sulfonated polysulfone and the electrostatic synergy of polydopamine (PDA). They reported that the introduction of β-CD and PDA changed the membrane’s surface potential, hydrophilicity, and pore structure. The application of such a membrane to remove Methylene blue from an aqueous solution enabled the removal of 97.8% of this dye. The obtained results indicate that modifying membrane pores with cyclodextrins has the potential to be a new research direction for the development of novel CD-based separation materials.

Recently, Zhou et al. [111] developed more complex, covalent organic frameworks (COFs)-polyester hybrid membranes via a simple one-step in situ co-polymerization method and used such novel materials for the separation of Congo red from sodium sulfate aqueous solution. In the synthesis procedure based on interfacial polymerization, they integrated p-phenylenediamine (Pa) and 1,3,5-triformylphloroglucinol (Tp) into a β-cyclodextrin (β-CD) aqueous solution and a trimesoyl chloride (TMC) organic solution, respectively. The β-CD and TMC underwent interfacial polymerization and formed a polyester layer; TpPa COFs were synthesized in this layer. The resulting complex membranes enabled efficient separation of dye (98.5%), were resistant to organic solvents, and had antifouling properties. Furthermore, the use of COFs in polyester membranes is a straightforward and cost-effective strategy that may become important in the future for the separation of dyes from dyeing wastewater. However, the obtained membrane was complex and contained several components, including a synthetic polymer.

In general, in the case of using cyclodextrins to produce membranes, one of the limitations is the need to also utilize other substances (e.g., to give the membranes the desired tensile strength), some of which cannot be considered environmentally friendly.

## 5. A Brief Summary of the Results Achieved Earlier Using Selected Polysaccharide-Based Membranes

Various membrane materials developed in recent years differ, often substantially, in relation to their composition, structure, and morphology, as well as to the efficiency and selectivity towards specific metal ions and dyes. However, because such separation materials were usually used under different experimental conditions (e.g., in simple, single-component or multi-component solutions or in real wastewater with a complex composition), it is not possible to clearly determine which membrane performed best. Selected examples of polysaccharide-based membranes (developed over the last few years), the use of which allowed efficient (>90%) separation of dyes and metal ions, have been presented in Table 3.

Research conducted in recent years related to the possibility of using various polysaccharides (e.g., alginates, cellulose and cyclodextrins) to produce membranes, intended for the separation of metal ions and dyes, has led to a deeper understanding of the factors influencing membrane processes. In the case of alginate-based membranes (e.g., those obtained using simple crosslinking), one of the main limitations of their use was their low mechanical strength and swelling in the solution. It has been shown that the mechanical properties of such materials (e.g., tensile strength) can be improved by introducing carefully selected additives, e.g., clays or carboxylated TiO_2_-COOH nanoparticles, which can be dispersed uniformly within the membrane [113,114]. However, due to the complexity of membrane processes, the possibilities of using different nanoparticles to produce mechanically strong, homogeneous membranes are still the subject of research [32,43]. Moreover, the results of many studies have shown that not only the type of polysaccharide used and the properties of other components of separation materials, but also their concentration had a significant impact on the efficiency of membrane processes [113,114,117,118]. Hence, many current studies are aimed at determining the optimal membrane composition for a given separation process [47,64,96,100]. The composition of membranes, both qualitative (e.g., type of polysaccharide and its origin) and quantitative, influences their structure (e.g., number, size and arrangement of pores), morphology and permeability. With respect to permeability, generally, it can be stated that highly permeable membranes have poorer performance. As can be seen from the data presented in Table 3, membranes of different compositions differed significantly in permeability. However, the efficiency of the polysaccharide-based membranes separation was also influenced by the solvents’ properties in which the membrane processes were carried out. It has been shown that, in the case of a cellophane material, various aprotic solvents (i.e., dimethylsulfoxide (DMSO), N-methylpyrrolidone (NMP), dimethylformamide (DMFA), tetrahydrofuran (THF), acetone) interacted differently with the membrane, which affected the swelling of cellulose (e.g., low degree of cellulose swelling has been observed in THF (37%) and high degree has been noted in DMSO (230%)). Moreover, it has been reported that the rejection of dyes (Orange II and Remazol Brilliant Blue) by the cellophane membrane correlated with the degree of cellulose swelling because polymer swelling has led to narrowing of the porous structure of the cellulose layer of the composite membrane (and consequently improved the efficiency of the separation processes) [116]. In summary, although it is impossible to determine which of the produced membranes performed best (as this depended on many factors, including the properties of the separated substances and the nature of the solution/suspension in which they occurred), it is possible, based on the available research results, to determine which factors significantly influenced the membrane processes. These include the appropriate material composition, which affected the structure and morphology of the membranes (e.g., a large number of regularly distributed pores of appropriate size, a large number of functional groups capable of binding the separated substances, low permeability), the appropriate selection of the membrane synthesis method (what had an impact on the material structure), and the use of appropriate conditions for conducting the membrane process, including the appropriate selection of solvents [39,116]. Despite how there are no universal, detailed guidelines regarding these factors (for example, with relation to membrane pore sizes, a size suitable for relatively small lithium ions separation may be inappropriate in the case of larger metal ions or dye molecules), based on the available research results, new “tailor-made” membranes can be designed, which, after optimizing all process conditions, can potentially perform even better.

## 6. Conclusions

The systematic growth of interest in environmentally safe methods intended for the separation of hazardous contaminants (e.g., synthetic dyes, heavy metal ions) from aqueous solutions is associated with the dynamic development of new membrane materials based on biodegradable and non-toxic polysaccharides and their derivatives. Recently, polysaccharides such as alginates, cellulose, starch, and chitosan [119,120] have been used as a basic membrane components, but due to the need to impart a range of essential properties to such materials (e.g., ability to selectively bind various contaminants, chemical resistance, antifouling properties and resistance to microorganisms), they were utilized much more often as one of the components in various types of more complex membranes.

The latest research directions in the development of such novel membranes include, among others, the possibility of using mixtures of various biopolymers (e.g., alginate and cellulose, alginate and carrageenan, starch and chitosan), biochar originated from various sources, and/or different MOFs and nanoparticles (e.g.,TiO_2_) to improve properties of these materials (e.g., to introduce more functional groups capable of binding specific contaminants, to increase the mechanical strength of the material). Efforts are also being made to obtain membranes with additional advantages, including high resistance to fouling, self-healing ability, and antibacterial properties, which is usually associated with the introduction of additional components to the material (e.g., addition of AgNPs in the case of antibacterial properties). However, incorporating specific additives into a membrane material typically solves a specific problem (e.g., related to limiting the growth of microorganisms on its surface), but often requires taking steps to address new limitations. For example, the use of silver nanoparticles in alginate-based hydrogel membranes imparts antibacterial properties, but the aggregation of AgNPs and their uneven dispersion throughout the material require the use of additional substances and steps in the synthesis process. Although research is mainly focused on developing well-performing membranes intended for separation of specific dyes and metal ions from various solutions, there are still many unresolved problems, e.g., related to the leaching of material components during membrane process, to long-term durability of membranes, or poor experimental data on the possibilities of their application on a larger scale. Generally, these problems apply (to a greater or lesser extent) to all types of polysaccharide-based membranes described.

Combining multiple components to produce membranes with desired properties often requires a rather complex synthesis procedure, although many studies have addressed the possibility of obtaining such materials using simple “one-pot” synthesis methods, characterized by relatively low costs. However, the formation of complex membranes suitable for effective separation of pollutants often requires the application of techniques such as grafting, crosslinking, or blending, which is associated with the need to use substances, not all of which can be described as eco-friendly. A material based on biodegradable biopolymers modified with chemical compounds (both, non-toxic and environmentally non-neutral) does not have to be environmentally safe and should be thoroughly tested, especially before being used on a larger scale. However, research on polysaccharide-based membrane properties related to their potential environmental impact (e.g., related to their toxicity, life-cycle assessment) is relatively scarce. Currently, research is also being carried out on the biodegradation processes (e.g., under the influence of fungi) of new polysaccharide-based membranes [121], but in the case of many developed new materials described in this review, such analyses are not yet a common solution.

Another issue related to novel polysaccharide-based membrane materials is the possibility of their reuse, regeneration, and disposal after they lose the ability to separate synthetic dyes and metal ions from aqueous solutions. While in most of the cited works, the authors attempted to reuse membranes in subsequent separation processes (with usually satisfactory but more or less decreasing efficiency), research on the possibility of their regeneration or the development of proper disposing methods of used materials is relatively rare.

An important issue in the context of using polysaccharide-based membranes is also estimating the costs of the synthesis and use of the membranes, including energy consumption. Although Authors of many studies often state that the developed method is “inexpensive” or “economical,” this information is usually not supported by specific calculations. Before using the most efficient and stable membrane materials for the separation of contaminants from aqueous solutions and suspensions on a wider scale, a clear estimation of all costs affecting the feasibility of the process will be necessary.

Despite the significant potential of polysaccharide-based materials in membrane processes, these problems need to be solved before novel complex membranes can be implemented on a larger (e.g., industrial) scale for the separation of synthetic dyes and metal ions from aqueous solutions.

## Figures and Tables

**Table 1:** Selected examples of the alginate-based hydrogel membranes intended for the separation of dyes, salts, and metal ions from various aqueous solutions.

Membrane Type and Composition	Separated Substances	Main Advantages of Novel Membrane Materials	Ref.
NaAlg-PVA-PVDF hydrogel composite nanofiltration (NF) membrane; sodium alginate and polyvinyl alcohol (PVA)—the hydrogel coatings, porous polyvinylidene fluoride (PVDF)—matrix	Methylene blue, Congo red, Coomassie brilliant blue	High retention ratios of 91.4% for Methylene blue, 95.6% for Congo red, 97.7% for Coomassie brilliant blue. Promising potential of membrane in treating printing and dye-laden wastewater.	[12]
CMCS-OA-NaAlg hydrogel composite membrane; sodium alginate and carboxymethyl chitosan (CMCS), non-metallic ions of oxalic acid (OA)—crosslinking agent	Right blue, Direct black, Direct red, Congo red, NaCl	The rejection higher than 95.0% for dyes and lower than 7.0% for NaCl. The membrane showed excellent anti-swelling and antifouling properties and performed well at high salt concentration.	[30]
OANaAlg/CMCS/TiO_2_@PTFE composite hydrogel membrane; CMCS, nano-titanium dioxide (TiO_2_), NaAlg aqueous solution (matrix), polytetrafluoroethylene (PTFE) nanofiber membrane—the support layer, OA—the crosslinking agent	Direct Black, Coomassie Brilliant Blue, Direct Red, Congo Red, NaCl, Na_2_SO_4_, MgCl_2_, MgSO_4_	High rejection rates: 99% for Direct Black, over 95% for all other separated dyes. Rejection rates below 10% for all inorganic salts. The membrane was characterized by very good mechanical properties (a tensile strength of 9.06 MPa), the embedded TiO_2_ could be recovered and reused. The membrane can be used for treating high-salinity wastewater under acidic conditions.	[31]
CuCaAlg/TiO_2_ hydrogel membranes; TiO_2_ nanoparticles, alginate polymer network, Ca^2+^/Cu^2+^—dual crosslinking agents	Coomassie brilliant blue, Direct black, Methyl orange, Congo red, Direct red, NaCl	The membrane exhibited excellent dye/salt selective separation performance (for Coomassie brilliant blue removal exceeded 99%, salt removal below 5%) and demonstrated excellent hydrophilicity, swelling resistance, and antifouling properties. Possibility of using a simple cleaning strategy (UV-H_2_O_2_-cross flow filtration) for rapid in situ cleaning of membrane in high-concentration dye solution.	[32]
P-MT@NaAlg-PVA-PVDF hydrogel nanofiltration (NF) membrane; multi-walled carbon nanotubes (MWCNTs) and TiO_2_ (P-MT)—coatings, NaAlg, polyvinyl alcohol (PVA), PVDF—matrix	Coomassie brilliant blue, Congo red, Methylene blue	High rejection rates: Coomassie brilliant blue—96.3%, Congo red—95.5%, Methylene blue—92.1%. Membrane displayed hydrophilicity and antifouling properties. It has great potential in the field of printing and dyeing wastewater treatment.	[33]
NaAlg/PEG/CNF/MWCNT-COOH hydrogel loose nanofiltration (LNF) membrane; NaAlg—matrix, large molecular weight PEG—pore-making agent, cellulose nanofibers (CNF) and carboxylated multi-walled carbon nanotubes (MWCNT-COOH)—additives	Crystal violet, Congo red, Tartrazine, Methylene blue, MgSO_4_, Na_2_SO_4_, MgCl_2_, NaCl	High rejection rates: Crystal violet—99.8%, Congo red—98.9%, Tartrazine—93.1%, Methylene blue—85.7%, low retention of salts (in the range of 7.3% to 11.6%). The membrane exhibited high separation efficiency towards the mixed dye/salt solution, showed excellent hydrophilicity and stain resistance, had good recycling effect	[34]
GO-CaAlg hybrid hydrogel membrane; graphene oxide (GO), calcium alginate hydrogel, Ca^2+^ ions—crosslinking agent	Coomassie brilliant blue, NaCl	Rejection rates: Coomassie brilliant blue—99%, NaCl—8%. The membrane maintained a good separation efficiency after 45 days of process, has shown superior antifouling performance, was characterized by antimicrobial properties (towards *E. coli*).	[35]
Car/CaAlg-NWF hydrogel composite filtration membrane; carrageenan (Car), calcium alginate, polyester NWF (nonwoven fabric)	Methylene blue	λ-Car/CaAlg-NWF exhibited superior dye rejection (100%). The hydrogel membranes were recyclable over nine cycles and have potential for water treatment applications.	[36]
NaAlg/CNF/UiO-66 dual-network composite hydrogel membrane; metal–organic frame UiO-66, sodium alginate, cellulose nanofibers (CNF)	Congo red Pb^2+^, Cu^2+^, Cd^2+^	Removal rates for Pb^2+^, Cu^2+^, and Cd^2+^ ions were 99.9%, 98.5%, and 96.5%, respectively. Removal rate for high concentration Congo red reached >99.8%. The membrane showed excellent reusability (high removal rates after ten consecutive filtration/elution cycles) and mechanical properties (resistant to deformation, the fracture stress exceeded 6 MPa), and may be, potentially useful in solving the problem of water resources pollution.	[37]
BCFS800/NaAlg composite hydrogel membrane; ball-milled crayfish shell biochar (BCFS800), sodium alginate, Ca^2+^ crosslinking agent	Congo red, NaCl	The membrane exhibited higher dye removal selectivity and lower inorganic salt ion rejection compared to the control membrane without biochar doping (149.2 vs. 34.3, and 4.54% vs. 7.56%, respectively), demonstrating exceptional dye/salt separation capability.	[38]
NaAlg/PVA hydrogel membrane; sodium alginate and poly(vinyl alcohol) (PVA)	Toluidine blue	The membrane (whose structure—pore size, quantity, and diameter distribution—depended on the number of freeze–thawing cycles used) showed significant potential for removing Toluidine blue dye from aqueous solutions with maximum adsorption capacity of 74.1 mg/g (Langmuir isotherm model).	[39]

**Table 2:** Selected examples of the cellulose and cellulose derivative-based membranes intended for the separation of dyes, salts, and metal ions from aqueous solutions.

Membrane Type and Composition	Separated Substances	Main Advantages of Novel Membrane Material	Reference/ Year of Publication
CNC-GLU TFN thin film nanocomposite membranes; cellulose nanocrystals (CNC) dispersed within a polyamide (PA) film, glutamic acid—modifier, polysulfone (PSF)	Methylene blue, Methyl orange, MgSO_4_, NaCl	CNC-GLU TFN membranes showed high dye rejection (99.83% for Methylene blue, 90.63% for Methyl orange) and salt removal (MgSO_4_ = 86%; NaCl = 38%). They demonstrated good operational stability after six cycles.	[56]
BiOCl/CNF/Mxene composite membrane; BiOCl, Mxene, cellulose nanofibers (CNF)	Congo red, Methyl blue, Rhodamine B, Crystal violet	Membrane showed excellent rejection for dyes (98.9% for Congo red, 97.2% for Methyl blue, 99.6% for Rhodamine B, 99.7% for Crystal violet), exhibited good stability and robust antifouling performance across different pH conditions.	[57]
PAF@BC multifunctional composite membrane; hydrophilic bacterial cellulose (BC), porous aromatic framework (PAF-45)	Anionic and cationic dyes, Iodide ions,	PAF@BC exhibited high efficiency and stability in intercepting both anionic and cationic dyes and iodide ions from aqueous solutions. The captured pollutants were effectively degraded. It also efficiently separated iodine vapor (96%). The membrane shows significant potential in environmental remediation and open new possibilities for the versatile application of composite membranes.	[58]
CA-NF-2/0.4 nanofiltration membranes; cellulose acetate (CA), polyamide (PA), diethylenetriamine (DETA), 1,3,5-benzenetricarbonyl chloride (TMC)	Rose Bengal, Congo red, Methyl orange, Methylene blue, MgCl_2_, Na_2_SO_4_, MgSO_4_, NaCl,	CA-NF-2/0.4 exhibited high removal efficiencies for dyes (99% for Rose Bengal and Congo red, 95.5% for Methyl orange, 96.1% for Methylene blue), and salts (MgCl_2_—84.2%, Na_2_SO_4_—92.7%, MgSO_4_—91.8%, NaCl—54.1%). Membranes demonstrated excellent antifouling properties (permeation recovery ratio >98% after three cycles of filtration), long-term durability, and stability (10 days) under high operational pressures and salt concentrations.	[59]
GC-CAMs gradient-charged membrane; cellulose acetate, ionic covalent organic framework nanosheets (iCOFNs), GC-gradient charged	Na_2_SO_4_, total dissolved solids,	GC-CAM achieved Na_2_SO_4_ rejection of about 95%, when applied to natural water purification, it reduced total dissolved solids to 83% while moderately removing heavy metal ions. The proposed gradient structure offers a novel approach for the charge engineering of nanofiltration membranes.	[60]
CA@TiO_2_ reverse osmosis composite membrane; CA, TiO_2_ nanoparticles, dimethylformamide (DMF)—solvent	Methylene blue, Ca^2+^, Mg^2+^, Ni^2+^, Zn^2+^, Cd^2+^, Cr^3+^, Fe^3+^,	The membrane showed over 60% of Ca^2+^and Mg^2+^ ions rejection, removed 90–97% of heavy metals ions, achieved about 85% oil/water separation efficiency and degraded 89.6% of Methylene blue under UV light in 90 min, showing good photocatalytic activity. The membrane offers promising potential for sustainable water purification and wastewater treatment.	[61]
MIL-125/CCNFs/PVDF multifunctional membrane; Ti-based MOF derived from Ti metal ions and carboxylate organic ligands (MIL-125), carboxylated cellulose nanofibers (CCNFs), PVDF	Crystal violet, Methylene blue, Malachite green,	MIL-125/CCNFs/PVDF exhibited excellent dye rejection (>97% for all dyes), strong self-cleaning ability, and antifouling stability. Developed separation method can be treated as a green and efficient strategy for next-generation water treatment.	[62]
EGO-DACNC-AgNPs composite membranes; ethylenediamine (EDA) and GO (EGO), silver nanoparticles (AgNPs), dialdehyde cellulose nanocrystal (DACNC)	Methylene blue, Crystal violet, Congo red	The membrane allowed for effective dye separation, had good mechanical stability, a long service life, strong antifouling properties. The degradation rate of Methylene blue (under visible light irradiation) reached 94% within 5 h, and the inhibition rate of *E. coli* reached 95%. Separation based on this type of membrane can potentially solve problems of membrane pollution and environmental protection in dye wastewater treatment.	[63]
Series of fully biobased membranes fabricated from pristine (P) and functionalized (T) cellulose nanofibers (CNF); T-CD-CNF-2,2,6,6-tetramethylpiperidin-1-oxyl (TEMPO), carbon dots (CDs), cellulose nanofiber (CNF), other membranes: T-CNF, P-CNF, CD-CNF	Protein (bovine serum albumin), Cu^2+^, Fe^+3^, Methylene blue	T-CD-CNF and T-CNF membranes have high filtration efficiency for heavy metals, dyes and protein from model solutions (e.g., for Methylene blue about 95% and 80%, respectively), in and garment industry wastewater (for MB about 20% and 25%, respectively). Such biobased composite membranes can be reused (in 5 cycles), which may have influence on the circular economy.	[64]
AA–IL-CA membranes; cellulose acetate, amino acids (AAs), 1-ethyl-3-methylimidazolium chloride ionic liquid (IL)	Fe^+3^, Pb^2+^, Zn^2+^, Cu^2+^,	The membrane enabled high rejection rates for copper, zinc, iron, and lead ions present in the industrial effluent (89%, 91%, 84%, and 90%, respectively). The use of CA, AAs, and IL aligns with sustainability goals; the cost-effectiveness, availability of materials, and the simplicity of the fabrication process suggest that the membrane fabrication process can be scaled up for large-scale industrial applications.	[65]
CTA/PVDF-HFP/TOA/TBP a blend polymer inclusion membrane (BPIM); cellulose triacetate (CTA), poly(vinylidene fluoride-co-hexafluoro propylene) (PVDF-HFP), tri-n-octylamine (TOA, extractant) and tri-n-butylphosphate (TBP, plasticizer)	Bi(III) in the presence of various metal ions, including Cu(II), Fe(III), Cr(III) Co(II), Zn(II), Pb(II), Cd(II), and Ni(II), Cd(II)	The BPIM exhibited selective extraction of Bi(III) (removal of 97%), in the presence of various metal ions, and was reusable (efficient in four consecutive cycles) BPIM is suitable for the efficient recovery of bismuth ions from zinc production plants residue.	[66]
CTA/AKL/D2EHPA polymer inclusion membranes; CTA, acetylated kraft lignin (AKL), di(2-ethylhexyl)phosphoric acid (D2EHPA)	Ni^2+^	The membranes were highly efficient in the recovery of Ni(II) ions (about 65% under optimized conditions).	[67]

**Table 3:** Selected examples of the polysaccharide-based membranes, developed over the last few years, intended for the separation of dyes, salts, and metal ions from aqueous solutions.

Membrane Composition/ Reference	Rejection/ Separated Substances	Selected Membrane Process Parameters	Additional Information
TiO_2_-COOH/CaAlg; carboxylated titanium dioxide and calcium alginate hydrogel nanofiltration membrane [112]	98.4% for Brilliant blue G250, 96.8% for Direct black, 38. 95.9% for Congo red	The permeate flux was about 14.1 L/m^2^⋅h	The membrane was characterized by low rejection ratios for inorganic salts (16.1%, 15.6%, 12.3% and 9.0% for MgSO_4_, Na_2_SO_4_, MgCl_2_ and NaCl, respectively).
Kaolin/CaAlg; kaolin, sodium alginate, Ca^2+^ (crosslinking agent), urea (porogen agent) [113]	100% for Brilliant blue G250, 95.2% for Congo red, 62.8% for Methylene blue, 99.7% for Cd^2+^	The permeate flux was 17.06 L/m^2^·h at 0.1 MPa.	Kaolin significantly improved the mechanical behavior of the membrane (with the 70% content of kaolin in NaAlg, the stress of the kaolin/CaAlg membrane reached 963.95 KP and was three times higher than in the case of CaAlg membrane).
GO/CEL; graphene oxide/cellulose, various coagulants [114]	100% for Co^2+^ 98–100% for Zn^2+^, 97–99% for Ni^2+^	The permeate flux was 50 L/m^2^⋅h,	The physicochemical properties of the coagulant used (e.g., molecular mass, dipole moment) had a large influence on the volume content of the membrane pores. In the case of 1-octanol GO/CEL membrane—volume fraction of pores was 1.82%
PDA/BNC; polydopamine (PDA) particles and bacterial nanocellulose (BNC) [115]	>98% in the case of Pb^2+^, Cd^2+^, Methyl orange, Methylene blue, Rhodamine 6G	-	The regenerated membranes exhibited excellent contaminant removal efficiency after 10 cycles of filtration (about 90%). The pore size of PDA/BNC was about 10 nm
Cellophane; cellulose solution in *N*-methyl- morpholine oxide, nonwoven polyester support [116]	15–85% for Orange II, 42–94% for Remazol Brilliant Blue R	Permeability depended on the solvent used (e.g., it changed from about 0.11 to about 2.5 kg/m^2^ h⋅bar, in the order: DMSO > NMP > DMFA > THF > acetone	Cellophane was stable in aprotic solvents, solvents interacted with the membrane material differently—a lower degree of cellulose swelling has been observed in THF (37%) and a higher degree has been noted in DMSO (230%). The rejection of solutes by the composite membranes correlated with the degree of cellulose swelling.
Nano-composite membranes CNCs, cellulose nanocrystals (CNCs) with different properties derived from raw microcrystalline cellulose [117]	99.9% for Methylene blue, 97.9% for Rhodamine B	The lowest permeate flux was about 12 L/m^2^⋅h	The permeation flux of the membranes depended on CNC concentration.
β -CD/TMC membranes, β -cyclodextrin and trimesoyl chloride (TMC) [118]	100.0% for Congo red, 99.1% for Rose bengal	A pure water flux of 207.8 L/m^2^⋅h at 2.0 bar	The membrane performance (e.g., permeate flux, efficiency) depended on β-CD and NaOH (aqueous additive) concentration. The rejection of NaCl was below 10.6%. The membrane showed desirable stability; the flux remained at 82.9% of the initial value after filtration for 16 h.

## Data Availability

The original contributions presented in this study are included in the article. Further inquiries can be directed to the author.
